# Recurrent acute myocardial and renal infarction with aplastic anaemia/paroxysmal nocturnal haemoglobinuria syndrome: a case report

**DOI:** 10.1093/ehjcr/ytae526

**Published:** 2024-09-23

**Authors:** Yuta Kato, Mitsuyoshi Hadase, Takashi Nakamura

**Affiliations:** Department of Cardiovascular Medicine, Saiseikai Shiga Hospital, 2-4-1 Ohashi, Ritto, Shiga 520-3046, Japan; Department of Cardiovascular Medicine, Saiseikai Shiga Hospital, 2-4-1 Ohashi, Ritto, Shiga 520-3046, Japan; Department of Cardiovascular Medicine, Saiseikai Shiga Hospital, 2-4-1 Ohashi, Ritto, Shiga 520-3046, Japan

**Keywords:** Acute myocardial infarction, Paroxysmal nocturnal haemoglobinuria, Aplastic anaemia, Multiple arterial thrombus, Anti-complement component C5 therapy, Case report

## Abstract

**Background:**

Aplastic anaemia (AA) is known to progress to paroxysmal nocturnal haemoglobinuria (PNH) during treatment, and thrombosis is a characteristic symptom of PNH. This case report investigates a case of repeated and rapidly progressive multiple arterial thrombosis due to PNH.

**Case Summary:**

This case is a 24-year-old woman undergoing treatment for AA. She presented with chest pain and underwent emergency coronary angiography. Thrombus occlusion was found in the distal portion of the right coronary artery, acute myocardial infarction was diagnosed and percutaneous coronary intervention was performed. Thrombus aspiration and balloon dilation were performed. Anticoagulants were administered, but chest pain flared up again on Day 9; coronary angiography was performed, indicating that the proximal portion of the right coronary artery had caused occlusion. On Days 9 and 24, she experienced back pain and was diagnosed with renal infarction. Considering that AA had evolved into PNH and intravascular haemolysis and thrombosis appeared, the diagnosis of PNH was made via flow cytometry. Multiple arterial thrombosis due to PNH was diagnosed, and ravulizumab treatment was started, resulting in the improvement of thrombus progression, chest pain, and back pain.

**Discussion:**

Thrombosis due to PNH can recur even after the administration of anticoagulants and antiplatelet agents and has been associated with a high fatality rate. The treatment with ravulizumab, a humanized monoclonal antibody against complement C5, helps with the prevention of thrombosis. Furthermore, anti-complement component C5 therapy is very effective in improving rapidly progressive multiple arterial thrombosis resistant to anticoagulants and antiplatelet agents due to PNH.

**Learning points**

- Aplastic anaemia can progress to paroxysmal nocturnal haemoglobinuria (PNH), and thrombosis caused by PNH could be lethal.
- PNH thrombosis is often resistant to anticoagulants and antiplatelet agents, and recurrent thrombosis has been reported.
- The development of anticoagulant-resistant thrombosis in patients with PNH could be fatal and requires early initiation of anti-complement component C5 therapy.

## Introduction

It is known that paroxysmal nocturnal haemoglobinuria (PNH) can develop during the treatment of aplastic anaemia (AA) and is referred to as AA-PNH syndrome. Moreover, PNH has three manifestations: intravascular haemolysis, thrombosis, and haematopoietic failure, of which thrombo-occlusion is a potentially lethal complication.^1^ Most cases of thromboembolism in PNH are venous thrombosis, with a small percentage of arterial thrombosis.^2^ Even in arterial thrombosis, myocardial infarction and multiple arterial thrombosis have been reported very infrequently.^3^ Thrombosis due to PNH is often resistant to anticoagulation, and anti-C5 humanized monoclonal antibodies such as eculizumab and ravulizumab have been proven to be effective.^4^

In this report, we presented a case of myocardial and renal infarction in which multiple anticoagulant-refractory thrombi appeared due to PNH during AA treatment and anti-complement component C5 therapy was found to be extremely effective.

## Summary Figure

**Figure:**
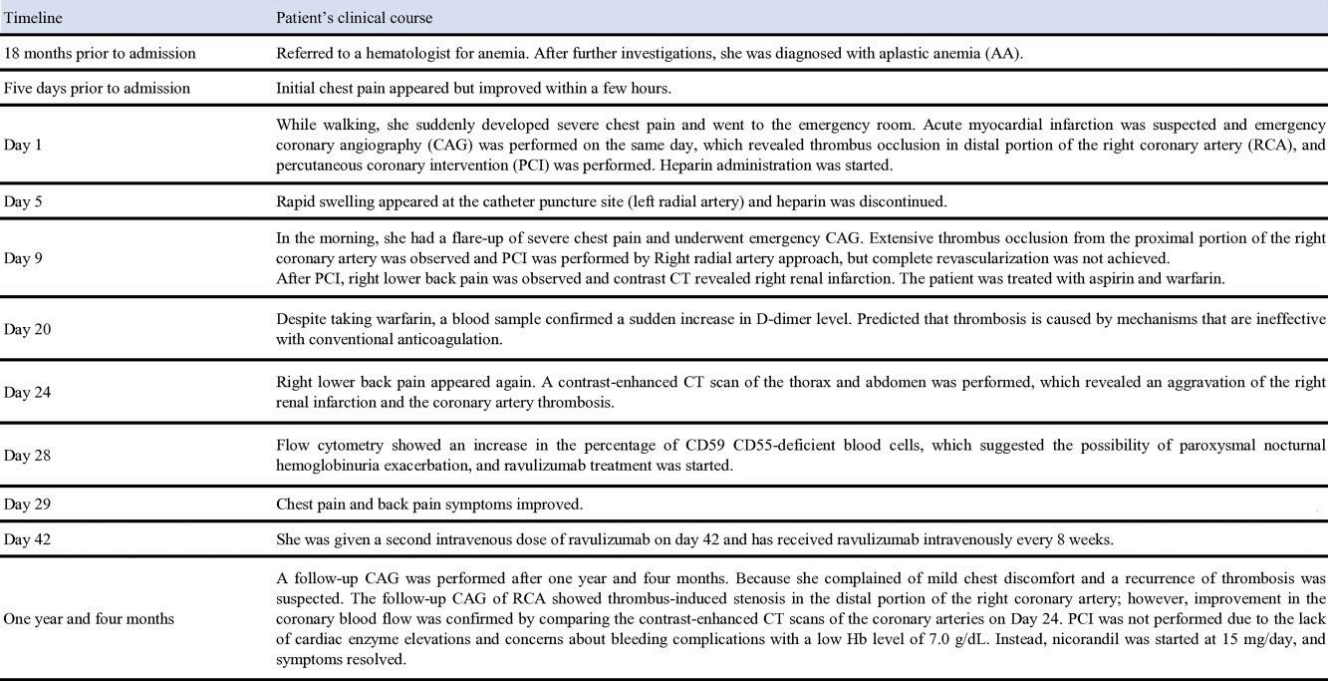


## Case presentation

**Figure 1:**
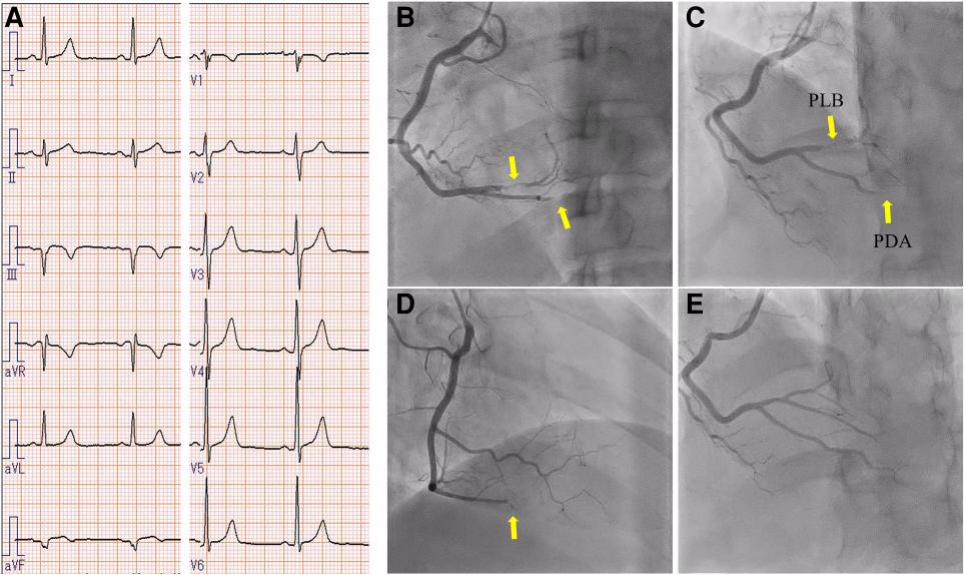
Electrocardiogram and first emergency coronary angiography and percutaneous coronary intervention (*A*) 12-lead electrocardiogram recorded before coronary angiography. (*B*)–(*D*) Right coronary arteries in emergency coronary angiography. The arrows indicate the areas of thrombus occlusion, and the branches of posterolateral branch and posterior descending artery appeared occluded. (*E*) Final coronary angiography of the right coronary arteries after percutaneous coronary intervention.

We present a case of a 24-year-old woman who had been diagnosed and treated for AA for 18 months. The patient was administered 150 mg cyclosporine and 100 mg danazol and was stable with no evidence of pancytopenia or haemolysis. Five days prior to her visit, she presented with symptoms of chest pain. On the day of her arrival, chest pain suddenly appeared while she was walking. She called emergency medical services as the chest pain was continuous and was accompanied by cold sweats. When she came to our hospital, her blood pressure was 125/70 mmHg, pulse rate was 64 b.p.m., saturation of percutaneous oxygen was 100%, heart sound was clear, and no murmur was audible. On admission, blood analysis revealed haemoglobin (Hb) of 14.2 g/dL (reference range, 11.6–14.8 g/dL), platelet count (PLT) of 91 000/µL (reference range, 158 000–348 000/µL), creatinine (CRE) of 0.71 mg/dL (reference range, 0.46–0.79 mg/dL), and creatinine kinase (CK) of 173 U/L (reference range, 59–248 mg/dL). An electrocardiogram showed ST-segment elevation in II, III, and aVF (*Figure 1A*), and a blood sample revealed elevated cardiac enzymes. An emergency coronary angiography (CAG) was performed to diagnose acute myocardial infarction, and emergency percutaneous coronary intervention (PCI) was performed because thrombus occlusion was observed in the posterior descending artery (PDA) and posterolateral branch (PLB) of the distal portion of right coronary arteries (RCA) (*Figure 1B–D*). Thrombus aspiration was performed to retrieve the thrombus, balloon dilatation was performed, and the procedure was completed after confirming the restoration of blood flow (*Figure 1E*;*Supplementary material online, Video 1*). The CK peaked at 1044 U/L on Day 2.

**Figure 2:**
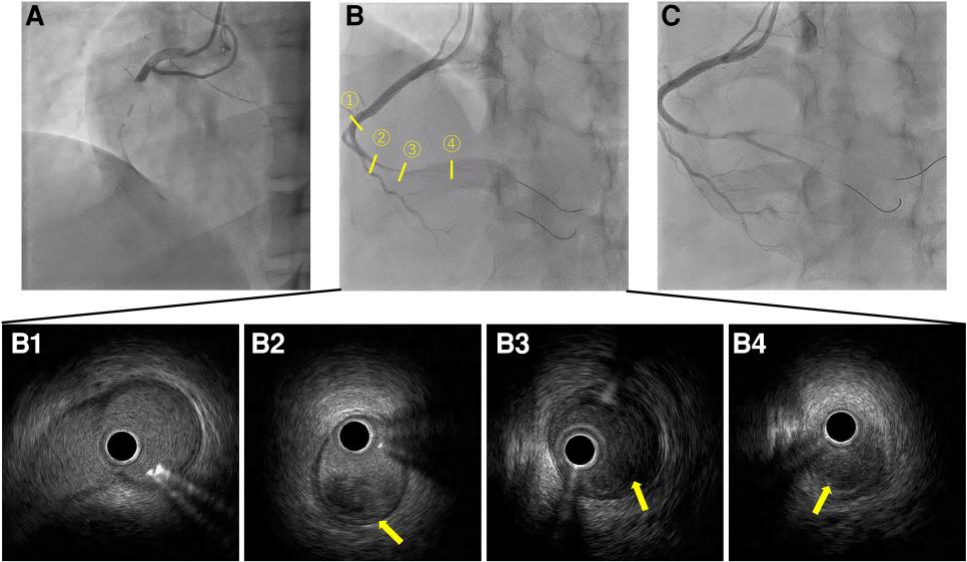
Second coronary angiography, percutaneous coronary intervention, and intravascular ultrasound (*A*) coronary angiography showed occlusion from the proximal portion of right coronary arteries, and no contrast of the distal right coronary arteries could be observed. (*B*) Coronary angiography and intravascular ultrasound findings after thrombus aspiration. The arrows on the intravascular ultrasound images indicate areas of low brightness within blood vessels, representing red thrombus. Large amounts of red thrombus filled the mid to distal portion of right coronary arteries. (*C*) Coronary angiography after percutaneous coronary intervention. Balloon dilation and thrombus retrieval were performed, but the thrombus was too massive for complete revascularization.

**Figure 3:**
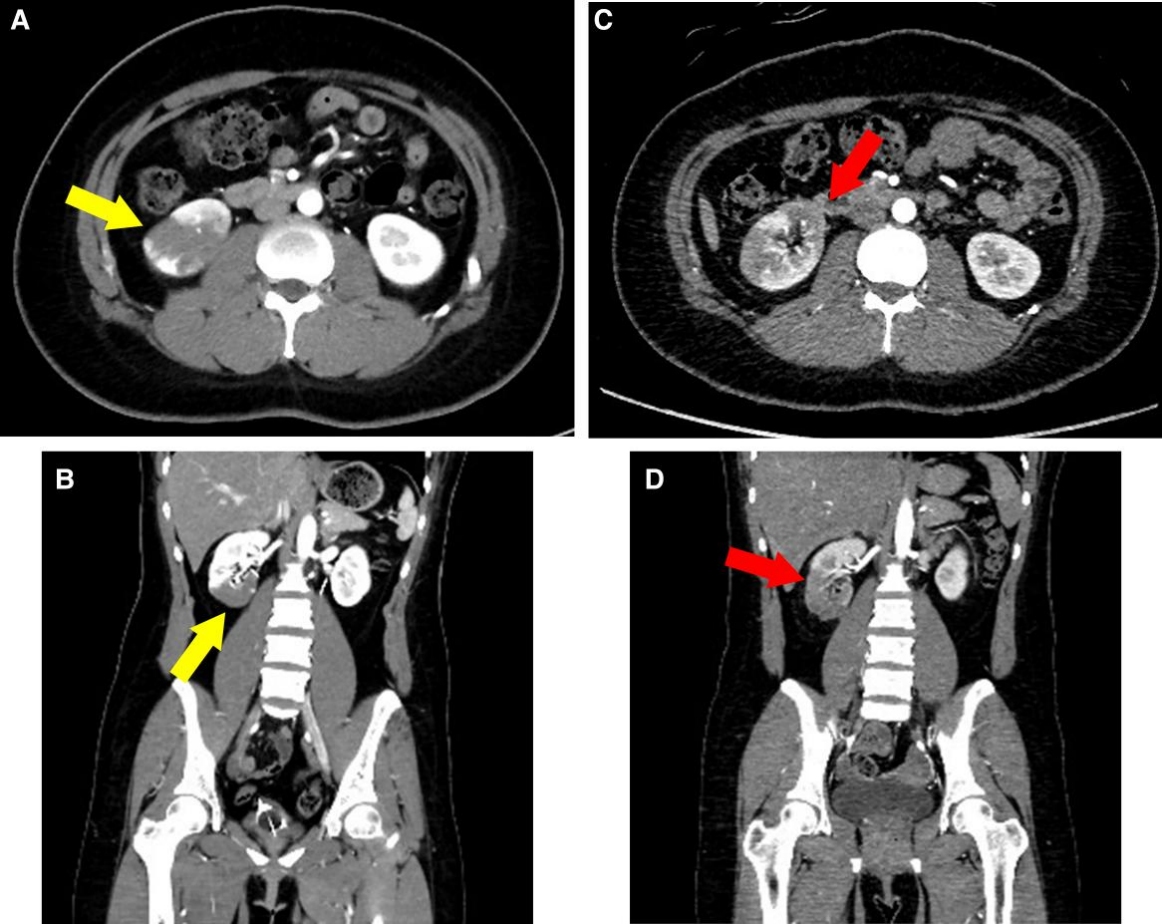
Contrast-enhanced computed tomography images (*A*, *B*) contrast-enhanced computed tomography scan on Day 9. Zonal contrast defect (arrows) in the right kidney, diagnosed as renal infarction. (*C*, *D*) Contrast-enhanced computed tomography imaging on Day 24 when the back pain flared up showed an exacerbation of the contrast defect (arrows) in the kidneys.

**Figure 4:**
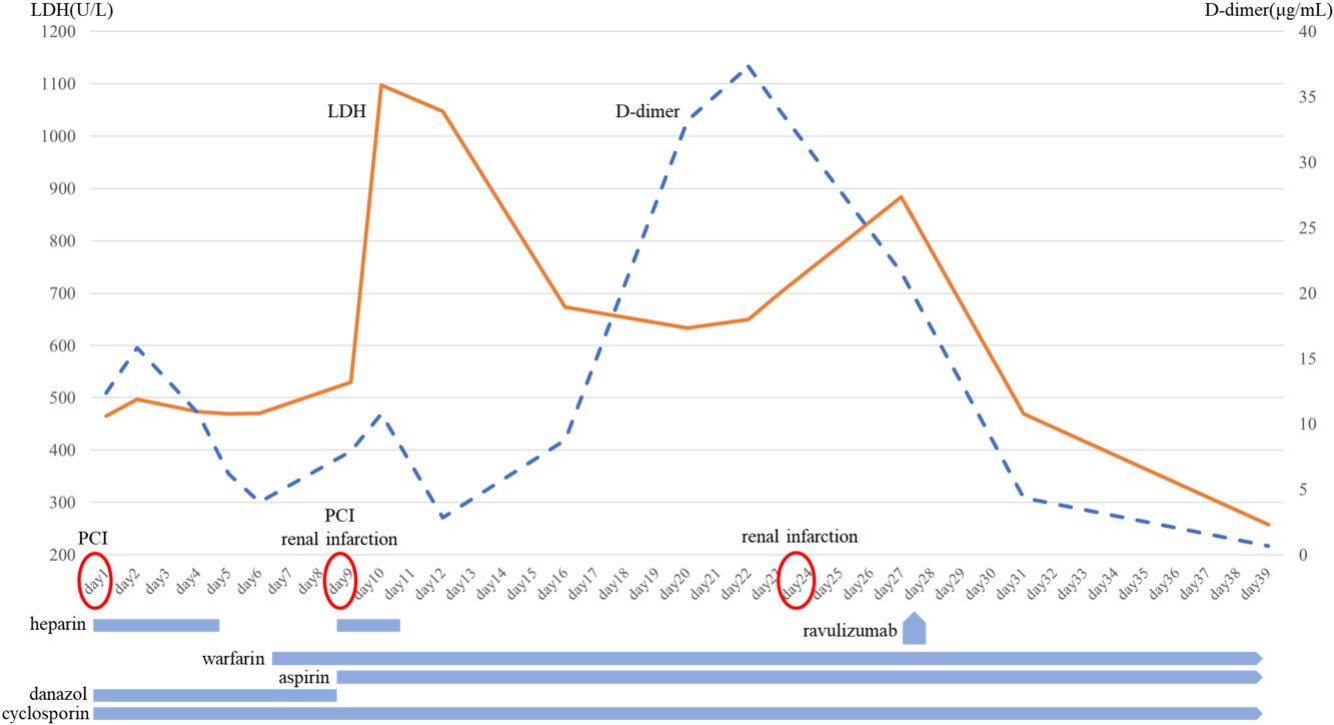
Clinical course graph showing D-dimer and lactate dehydrogenase transitions in the blood sampling tests. A rapid increase in the lactate dehydrogenase level after the appearance of renal infarction and a significant increase in the D-dimer level despite the anticoagulant medication. The patient was judged to be affected by PNH and was treated with ravulizumab, which rapidly reduced the levels of lactate dehydrogenase and D-dimer. Changes in antiplatelet drugs, anticoagulants, and drugs used for aplastic anaemia are listed below the graph. PNH, paroxysmal nocturnal haemoglobinuria.

**Figure 5:**
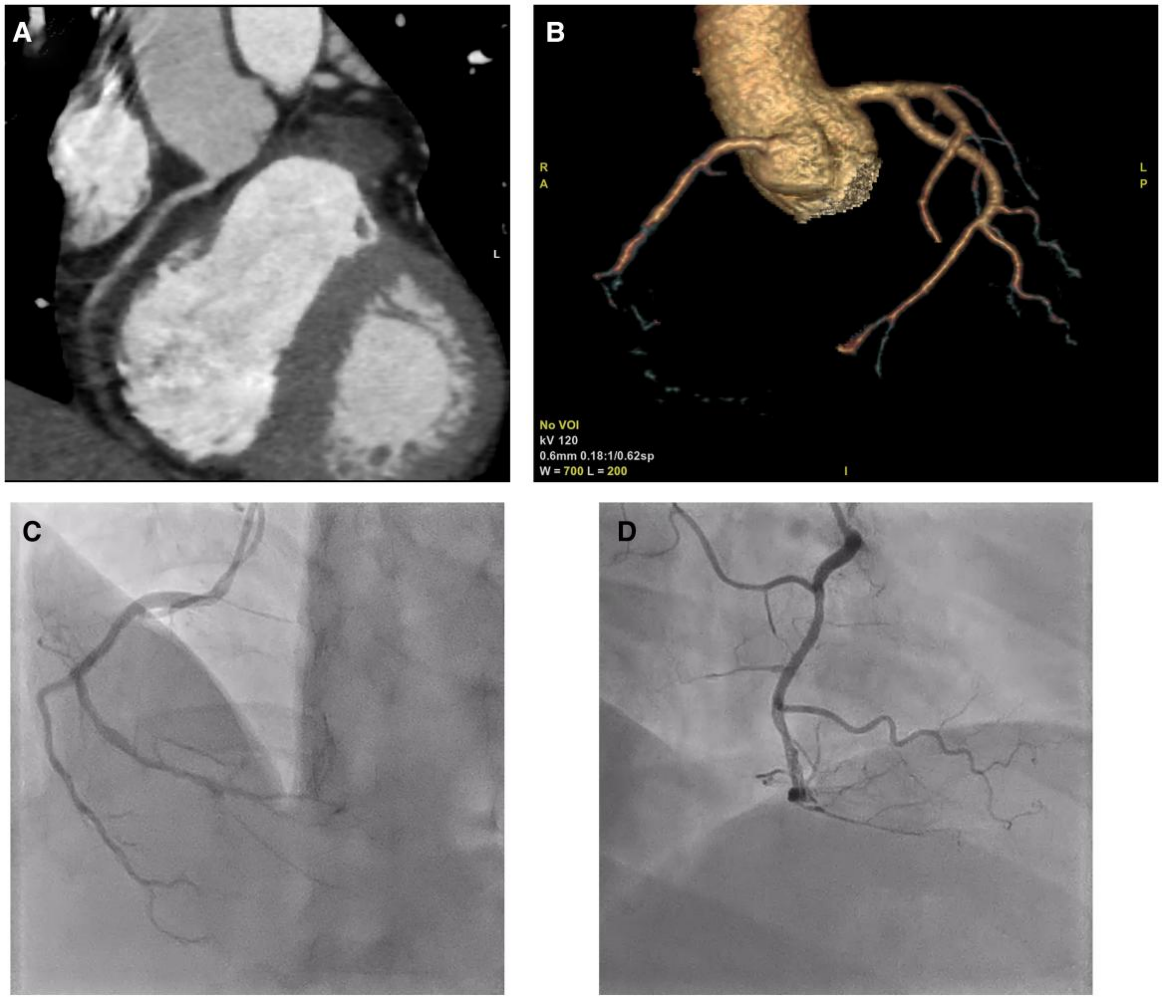
Follow-up coronary angiography of right coronary arteries after 1 year and 4 months (*A*, *B*) contrast-enhanced computed tomography of right coronary arteries on Day 24. Percutaneous coronary intervention was performed on Day 9, but thrombotic occlusion recurred from the proximal right coronary arteries on Day 24. (*C*, *D*) Coronary angiography of the right coronary arteries. An improved coronary blood flow was confirmed via coronary angiography when compared with the contrast-enhanced computed tomography of the coronary arteries performed on Day 24.

As the patient had a non-atherosclerotic myocardial infarction caused by a thrombus, heparin was administered intravenously at a rate of 10 U/kg/h. On Day 5, her forearm began to swell rapidly, resulting in bleeding. Blood analysis revealed a Hb of 12.0 g/dL and PLT of 43 000/µL. The patient was found to have an easy bleeding tendency due to low platelets caused by AA. Heparin administration ended on Day 5, and warfarin was started on Day 7 after confirmation of improvement in forearm bleeding. On Day 9, she complained of severe chest pain with cold sweats. Blood analysis revealed on Day 6 showed a CK of 156 U/L, which increased to 293 U/L on Day 9. Emergency CAG was performed again, which revealed a massive thrombus occlusion in the proximal to distal portion of RCA (*Figure 2A*). The vessels were observed by intravascular ultrasound (IVUS), which showed a large amount of red thrombus (arrows) filling in the coronary arteries (*Figure 2B*). Intravascular ultrasound excluded plaque rupture and proved embolization as the cause of the coronary obstruction. Repeated thrombus aspiration and balloon dilatation were performed, but complete peripheral revascularization could not be achieved (*Figure 2C*;*Supplementary material online, Video 1*). The patient had AA and bleeding complications that were deemed potentially fatal. Stenting would have required short-term dual antiplatelet therapy and anticoagulants, which could have exacerbated the bleeding. Therefore, we decided against stent placement in the right coronary artery. The patient was started on aspirin due to recurrent arterial thrombosis, which recurred on anticoagulants alone. However, the patient did not have a coronary stent implanted and, therefore, opted for aspirin and warfarin treatment instead of dual antiplatelet therapy. Danazol was discontinued due to thrombophilia, and the dose of warfarin was adjusted to achieve an effective blood concentration of about International normalized ratio 2.0–2.5. On the same day, the patient complained of severe right lower back pain with cold sweats, and a contrast-enhanced computed tomography (CT) scan revealed a right renal artery infarction (*Figure 3A and B*). Moreover, lactate dehydrogenase (LDH) increased significantly after renal infarction (*Figure 4*). The blood analysis on Day 10 showed that Hb dropped to 10.4 g/dL, PLT to 56 000/µL, and CK peaked at 403 U/L. CRE was 0.75 mg/dL, and there was no exacerbation. Although a thorough examination for thrombogenicity was conducted in parallel, tumour markers and heparin-induced thrombocytopenia antibodies were negative. Screening for congenital predisposition to thrombosis was also tested but found to be negative, and no obvious cause was identified through blood sampling. The echocardiography of the veins of the lower extremities did not reveal deep venous thrombosis (DVT), contrast-enhanced CT did not reveal any thrombotic lesions other than renal infarction, and the head MRI did not reveal cerebral infarction. The transoesophageal echocardiography did not show any intracardiac thrombus or left–right shunt, and the pathological examination of the thrombus specimen obtained during PCI did not detect any malignant tumours. A systemic search was performed, but no clots were found except in the coronary arteries and kidneys. On Day 16, a blood sample showed Hb of 7.6 g/dL and PLT of 93 000/µL, indicating anaemia progression. On Day 20, a blood sample showed a sharp increase in the D-dimer level despite warfarin administration (*Figure 4*). At this point, we assumed that the cause of thrombosis was a mechanism that would not respond to conventional anticoagulant therapy. In particular, thrombosis due to haemolysis and other factors was suspected due to the persistent elevation of LDH. On Day 24, the patient again complained of right lower back pain, and a blood sample on the same day showed a CRE of 0.93 mg/dL, indicating worsening renal function. As recurrence of thrombosis was suspected, a contrast-enhanced CT scan of the thorax and abdomen was performed, which revealed aggravation of the right renal infarction (*Figure 3C and D*) and coronary artery thrombosis (*Figure 5A and B*). Considering the progression of anaemia, it was decided that any further administration of anticoagulants or antiplatelet medication would be fatal. Moreover, the patient did not present with any new chest complaints and no additional treatment was given.

It is known that PNH can occur during the treatment of AA. She found that her morning urine was brownish, and gross haemoglobinuria was suspected. The flow cytometry showed a prominent increase in CD59 CD55-deficient blood cells. A series of symptoms were diagnosed as intravascular haemolysis and thrombosis due to PNH. The first intravenous dose of ravulizumab (2700 mg) was started on Day 28, which significantly reduced D-dimer and LDH and improved the symptoms (*Figure 4*). Since the chest symptoms and blood tests improved, she was discharged from the hospital on Day 39. The blood sample at discharge showed Hb of 10.8 g/dL and PLT of 62 000/µL. Intravenous ravulizumab (3300 mg) was administered 2 weeks after the first dose, followed by a similar dosage every 8 weeks thereafter. A follow-up contrast-enhanced CT examination after 6 months revealed an improvement in RCA thrombosis and renal infarction with no observed recurrence of thrombosis. A follow-up CAG was performed after 1 year and 4 months, when she complained of mild chest discomfort. The follow-up CAG of RCA showed thrombus-induced stenosis in the distal portion of the right coronary artery; however, improvement in the coronary blood flow was confirmed by comparing the contrast-enhanced CT scans of the coronary arteries on Day 24 (*Figure 5A–D*). PCI was not performed due to the lack of cardiac enzyme elevations and concerns about bleeding complications with a low Hb level of 7.0 g/dL. Instead, nicorandil was started at 15 mg/day, and symptoms resolved. To date, we are prescribing aspirin and warfarin as antithrombotic agents, cyclosporine for AA, and intravenous ravulizumab for PNH. As a result, Hb improved to 14.0 g/dL, PLT to 220 000/µL, and LDH to 220 U/L (reference range, 124–222 U/L), with no evidence of anaemia or haemolysis.

## Discussion

AA is a syndrome characterized by pancytopenia in the peripheral blood and hypoplasia of the bone marrow. It is known that PNH can occur during the treatment of AA. PNH is known to have three manifestations: intravascular haemolysis, thrombosis, and haematopoietic failure, of which thrombo-occlusion is a potentially lethal complication. In Western countries, 35% of patients with PNH were reported to die within 5 years of diagnosis^1^; the rate of occurrence of thrombosis in PNH is 40%, and venous and arterial thrombosis account for ∼40–67% of PNH-related deaths.^2^ Venous thrombosis accounts for 85% of thrombosis, and arterial thrombosis 15%. Furthermore, the rate of myocardial infarction and unstable angina is found to be extremely low at 1.6%.^2^

PNH is a disease wherein the loss of Glycosylphosphatidylinositol anchored membrane protein results in uncontrolled complement activation and intravascular haemolysis, which can lead to organ damage and thrombosis. The C3 and C5 are activated, and a complement complex is formed. The attack of this complement complex on red blood cell membranes causes intravascular haemolysis. Although the pathogenesis of thrombosis has not been fully investigated, it is believed that free Hb generated by intravascular haemolysis may cause thrombus formation, either directly or via nitric oxide absorption. Platelet activation due to CD59 deficiency, impaired fibrinolytic system, and inflammatory mediators have also been implicated, which suggests that multiple factors might contribute to thrombosis in patients with PNH. Moreover, thrombosis itself leads to complement activation, which in turn leads to further thrombosis, a vicious cycle that continues. Once a patient develops an initial thrombosis, further thrombotic complications are considered the cause of uncontrollable complications despite anticoagulation therapy.^4^ Thrombosis due to PNH can recur even with anticoagulants and antiplatelet agents, and a high fatality rate due to thrombosis has been reported.^2^ Some studies have reported acute in-stent thrombosis appearing after myocardial infarction, while the patient was on dual antiplatelet therapy and heparin.^3^

However, the development of an anti-C5 humanized monoclonal antibody (eculizumab and ravulizumab) allows the prevention of recurrent thrombosis.^4^ The 5-year survival rate for patients not treated with eculizumab was 66.8%, whereas the 5-year survival rate for patients treated with eculizumab was 95.5%.^5^ The development of thrombosis in patients with PNH is now considered one of the primary indicators for initiating anti-complement component C5 therapy, which should be performed without delay.

The gold standard test for PNH is the flow cytometry of red blood cells. Increased PNH clones are a known indicator of risk assessment for thrombosis. Patients with PNH and granulocyte clones (> 50%) were reported to have a 44% risk of venous thrombosis over 10 years, with 5.8% of patients with smaller clone sizes having venous thrombosis.^6,7^ Elevated LDH, reticulocytes, and haemoglobinuria are highly indicative of PNH.

In this case, the blood samples were taken regularly, and although LDH was higher than normal, no evidence of a rapid increase that would suggest a haemolytic process but rather a rapid increase after the appearance of the initial myocardial infarction was observed. Although findings of haemolysis, such as anaemia and elevated LDH, had already been observed, the diagnosis was delayed because we believed that the anaemia progression was due to bleeding complications and the elevated LDH was due to post-myocardial infarction or post-renal infarction. In the beginning, *in situ* thrombosis in the coronary and renal arteries appeared due to mild intravascular haemolysis, but the patients may not be aware of any symptoms. Subsequently, we believed that the thrombus itself had triggered complement activation and further thrombosis. Therefore, the patients considered the possibility of repeated thrombosis at the same site.

Thrombosis improved after ravulizumab treatment compared with anticoagulants treatment, which suggested that the anti-complement component C5 therapy should be administered immediately if fatal thrombosis is observed in patients with PNH, even if the LDH levels are mild.

## Conclusion

This report presents a case of myocardial and renal infarction in which multiple anticoagulant-refractory thrombi were observed due to PNH during AA treatment. The findings of this case reveal that ravulizumab treatment was extremely effective.

## Lead author biography


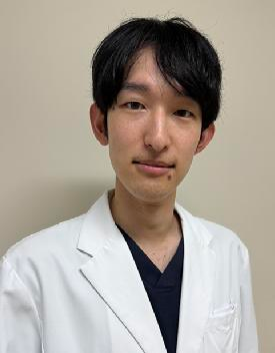


Dr Yuta Kato graduated from Kyoto Prefectural University of Medicine in 2020. He is currently working in the Department of Cardiology, Saiseikai Shiga Hospital, in Shiga, Japan, specializing in coronary intervention, peripheral vascular intervention, and heart failure.

## Supplementary Material

ytae526_Supplementary_Data

## Data Availability

The data underlying this article are available in the article and in its online Supplementary material.

## Notes

**Consent:** The authors confirm that written consent for submission and publication of this case report including images and associated text has been obtained from the patient in line with COPE Guidance.

**Funding:** No funding to be declared.
