# Supplementary material for: Recurrent acute myocardial and renal infarction with aplastic anaemia/paroxysmal nocturnal haemoglobinuria syndrome: a case report
Source: Eur Heart J Case Rep. 2024 Sep 23;8(10):ytae526. doi: 10.1093/ehjcr/ytae526 (PMC11462453; doi:10.1093/ehjcr/ytae526)
Supplement: ytae526_Supplementary_Data [file ytae526_supplementary_data.zip › renamed_b3481.pdf]

Crimson | ulatus Translation | voxtab Transcription | enago English Editing

CERTIFICATE OF EDITING

This is to certify that the paper titled "**Rapidly progressive acute myocardial and renal infarction with aplastic anemia/paroxysmal nocturnal hemoglobinuria syndrome: a case report"** commissioned to us has been edited for English language, grammar, punctuation, and spelling by Enago, the editing brand of Crimson Interactive Pvt. Ltd under **Advance Editing**.

Issued by:
Enago, Crimson Interactive Pvt. Ltd
1001, Techniplex - II, S. V. Road,
Goregaon (W), Mumbai 400062, India.
Phone: 03-5050-5374
Fax: 03-4496-4934

Disclaimer: The author is free to accept or reject our changes in the document after our editing. However, we do not bear responsibility for revisions made to the document after our edit on 30th March, 2024**.**

Global	www.enago.com, www.voxtab.com, www.ulatus.com
Japan	www.enago.jp, www.ulatus.jp, www.voxtab.jp
Taiwan	www.enago.tw
China	www.enago.cn
Brazil	www.enago.com.br
Germany	www.enago.de
Russia	www.enago.ru
Arabic	www.enago.ae
Turkey	www.enago.com.tr
S. Korea	www.enago.co.kr

About Crimson:
Crimson Interactive Inc. provides English language editing, transcription, and translation services to individuals and corporate customers worldwide.
